# Psychoeducational Messaging to Reduce Alcohol Use for College Students With Type 1 Diabetes: Internet-Delivered Pilot Trial

**DOI:** 10.2196/26418

**Published:** 2021-09-30

**Authors:** Lauren E Wisk, Kara M Magane, Eliza B Nelson, Rebecca K Tsevat, Sharon Levy, Elissa R Weitzman

**Affiliations:** 1 Division of General Internal Medicine and Health Services Research David Geffen School of Medicine University of California Los Angeles Los Angeles, CA United States; 2 Division of Adolescent/Young Adult Medicine Boston Children's Hospital Boston, MA United States; 3 Department of Pediatrics Harvard Medical School Boston, MA United States; 4 Division of Developmental Medicine Boston Children's Hospital Boston, MA United States; 5 Computational Health Informatics Program Boston Children’s Hospital Boston, MA United States

**Keywords:** adolescent, young adult, diabetes mellitus, type 1, binge drinking, alcohol drinking, self care, risk-taking, universities, students, attitude, mobile phone

## Abstract

**Background:**

College environments promote high-volume or binge alcohol consumption among youth, which may be especially harmful to those with type 1 diabetes (T1D). Little is known about the acceptability and effectiveness of interventions targeting reduced alcohol use by college students with T1D, and it is unclear whether intervention framing (specifically, the narrator of intervention messages) matters with respect to affecting behavior change. Interventions promoted by peer educators may be highly relatable and socially persuasive, whereas those delivered by clinical providers may be highly credible and motivating.

**Objective:**

The aim of this study is to determine the acceptability and impacts of an alcohol use psychoeducational intervention delivered asynchronously through web-based channels to college students with T1D. The secondary aim is to compare the impacts of two competing versions of the intervention that differed by narrator (peer vs clinician).

**Methods:**

We recruited 138 college students (aged 17-25 years) with T1D through web-based channels and delivered a brief intervention to participants randomly assigned to 1 of 2 versions that differed only with respect to the audiovisually recorded narrator. We assessed the impacts of the exposure to the intervention overall and by group, comparing the levels of alcohol- and diabetes-related knowledge, perceptions, and use among baseline, immediately after the intervention, and 2 weeks after intervention delivery.

**Results:**

Of the 138 enrolled participants, 122 (88.4%) completed all follow-up assessments; the participants were predominantly women (98/122, 80.3%), were White non-Hispanic (102/122, 83.6%), and had consumed alcohol in the past year (101/122, 82.8%). Both arms saw significant postintervention gains in the knowledge of alcohol’s impacts on diabetes-related factors, health-protecting attitudes toward drinking, and concerns about drinking. All participants reported significant decreases in binge drinking 2 weeks after the intervention (21.3%; odds ratio 0.48, 95% CI 0.31-0.75) compared with the 2 weeks before the intervention (43/122, 35.2%). Changes in binge drinking after the intervention were affected by changes in concerns about alcohol use and T1D. Those who viewed the provider narrator were significantly more likely to rate their narrator as knowledgeable and trustworthy; there were no other significant differences in intervention effects by the narrator.

**Conclusions:**

The intervention model was highly acceptable and effective at reducing self-reported binge drinking at follow-up, offering the potential for broad dissemination and reach given the web-based format and contactless, on-demand content. Both intervention narrators increased knowledge, improved health-protecting attitudes, and increased concerns regarding alcohol use. The participants’ perceptions of expertise and credibility differed by narrator.

**Trial Registration:**

ClinicalTrials.gov NCT02883829; https://clinicaltrials.gov/ct2/show/NCT02883829

**International Registered Report Identifier (IRRID):**

RR2-10.1177/1932296819839503

## Introduction

### Background

Effective management of chronic conditions such as type 1 diabetes (T1D) is affected by individual-, family-, community-, and health care system–level features [1]. For adolescents and young adults with T1D who may struggle to meet glycemic targets [2], disease self-management (eg, blood glucose monitoring) and risk behaviors (eg, alcohol consumption and poor diet) are some of the many factors that influence the achievement of glycemic targets [3,4]. The transition to college may be a particularly vulnerable period, as self-management processes are disrupted, risk behaviors rise, and access to support and supervision from family and health care providers diminishes [5].

Clinical studies in the United States have found that 1 in 3 youths with T1D may drink alcohol by the time they reach high school [6]. Postsecondary education (eg, college or university) students with T1D may be especially vulnerable to initiating or intensifying alcohol use, as many reside in settings that are ill-prepared to identify and support their health needs [7]. *Alcogenic* college environments [8-10] create social pressures that foster alcohol use [11,12]. Despite the risks associated with alcohol consumption and T1D [13,14], college-aged youths with T1D have poor alcohol-related health literacy [15,16] and engage in risky alcohol use [8,17]. There is a dearth of evidence-based interventions to minimize alcohol-related risks in youths with T1D in general [3,16,18] and fewer targeting those in college [19].

Given the high potential for alcohol-related health harm, it is critically important to identify how best to engage college students with T1D around harm reduction [20]. Consistent with the students’ developmental status, successful interventions may require a patient-centered focus and peer or provider directives, reinforcing the value of health-protecting behavior [21-24]. A digital approach to delivering specialized information may be especially advantageous in situations where college students are distanced from their specialty care or where college health services may be underresourced for the provision of chronic disease–specific guidance [25]. The digitally oriented nature of this age group suggests that the students will be highly receptive to such delivery [26]; however, even digital materials may benefit from delivery by a compelling spokesperson who can appropriately frame the educational materials. Communication sciences and behavioral economics posit different ways in which the presentation of information can affect decision-making; thus, understanding the acceptability and impact of different narrators for delivering harm reduction messages may optimize their effects. Peer delivery could be especially meaningful if college students with T1D personally identify with the narrator and feel supported regarding the distress arising from contravening social norms that encourage drinking. Conversely, provider delivery may be highly salient—clinician expertise, gravitas, and the grounding of guidance in diabetes science may be impactful for shaping health beliefs and behaviors.

### Objective

The aims of this study are to understand the acceptability of a digital health intervention for addressing alcohol use risk among college students with T1D and to test the relative salience of two types of narrators for its delivery and framing. Consistent with a stepped approach for designing behavioral interventions [27,28], we undertook a pilot trial to evaluate the acceptability and impacts of the intervention overall and measure the relative salience of competing versions of the intervention, with one version delivered by a peer educator and another delivered by a specialty care provider (ie, a medicine-pediatric endocrinologist). The intervention coupled diabetes-related medical science and social-emotional content, building on formative work indicating that both elements are considered high priorities for substance use–related interventions targeting adolescents and young adults with chronic conditions [22]. Given prior evidence about the effectiveness of peer support for diabetes self-management [29-34], we hypothesized that exposure to peer delivery of this information would show higher acceptability and impacts on knowledge, attitudes, and behaviors than provider delivery.

## Methods

### Overview

We implemented a pilot trial to test two competing versions of a novel psychoeducational intervention targeting alcohol-related knowledge, attitudes, and behaviors of college students with T1D from the United States and Canada. The approach to engaging participants has been previously reported [35]. For the pilot, we randomized the narrators delivering and framing the educational materials. Both structured and open-ended survey data were collected. This study was approved by the institutional review board.

### Recruitment and Consent

**Figure 1:**
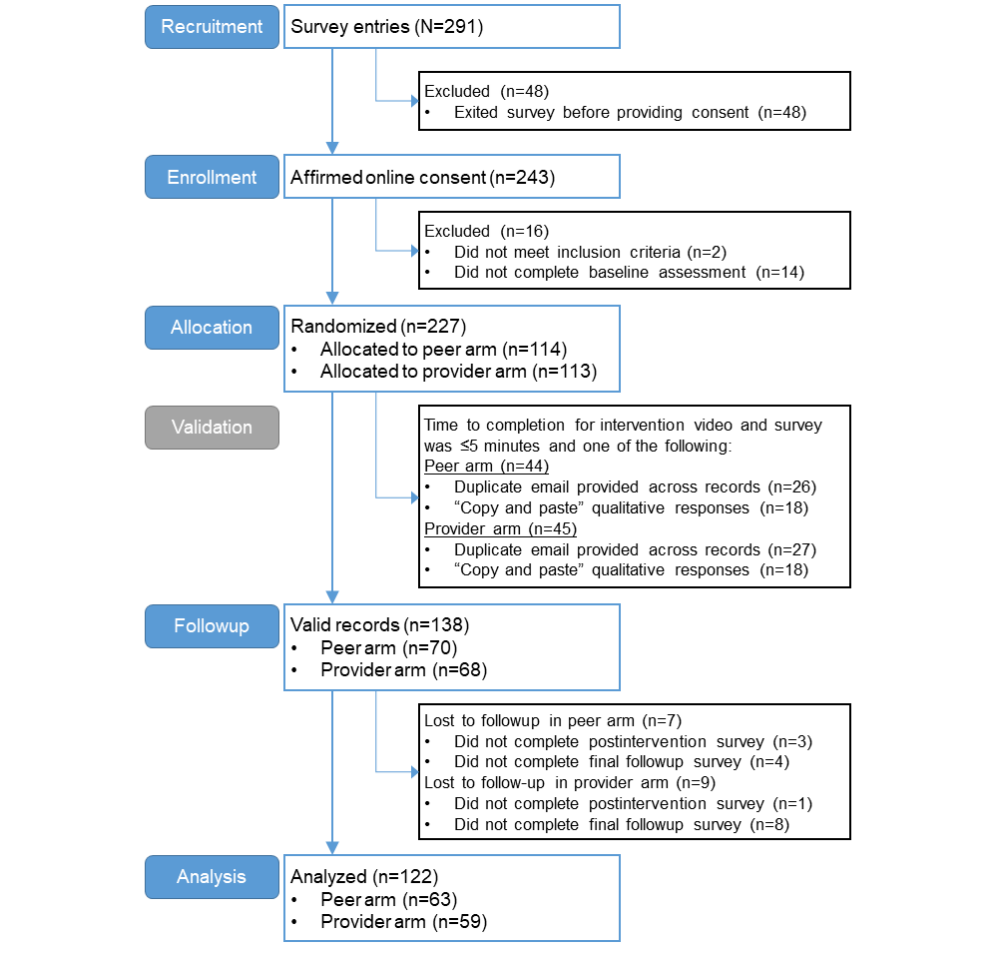
Trial CONSORT (Consolidated Standards of Reporting Trials) diagram showing participant flow through each stage of the randomized trial from recruitment through analyses.

The participants were recruited from two diabetes advocacy groups through social media, direct email newsletters, and a website banner. The recruitment messages were linked to a website with additional study details and safety resources. The website, in turn, transferred interested individuals to a REDCap (Research Electronic Data Capture) survey hosted on secure servers; the website and survey were accessible on a smartphone, tablet, or computer. After entering the survey, the individuals completed the web-based informed consent (or assented with a waiver of parental consent for those aged 17 years). Additional details of recruitment procedures and study implementation, including an evaluation of sampling procedures and generalizability, have been previously published [35] and are summarized in the CONSORT (Consolidated Standards of Reporting Trials) diagram (Figure 1).

After providing consent, the respondents answered screening questions to ascertain eligibility (aged 17-25 years, received a diagnosis of T1D, and currently attending, enrolled at a college, or university), and those who met the inclusion criteria (hereafter *participants*) were directed to complete the remainder of the baseline survey.

### Intervention and Assessments

Upon completion of the baseline survey, the participants were automatically randomized to receive one of two brief psychoeducational videos narrated by either an endocrinologist (provider) or a college student with T1D (peer educator). A REDCap module executed stratified randomization based on sex, year in college, and alcohol use in the past year. The intervention video included educational content about the effects of alcohol on physical and psychological functioning in persons with T1D and addressed the social aspects of alcohol use through visually rich, annotated graphics that described how and why youths with T1D choose to limit alcohol use in social settings; overall, the video had a harm reduction rather than abstinence focus that did not specifically call for abstinence or suggest safe limits in use.

To develop the psychoeducational content for the intervention, we conducted qualitative interviews using a semistructured interview guide grounded in social cognitive theories of risk-taking among adolescents with chronic conditions [22]. The topics covered daily experiences of the patient’s medical condition; information about personal, social, situational, and clinical factors affecting decision-making regarding alcohol use; and preferences for clinical communication about substance use. The results of the qualitative interviews, in combination with prior epidemiological investigations to ascertain salient concerns, motivations, and consequences of alcohol use [6,9,22,36-38], informed the development of an intervention containing patient-centered, accessible information regarding disease-specific concerns and goals while also addressing the complexity of navigating chronic disease management and alcohol use in the setting of adolescent-typical peer pressures and impulses. Clinically accurate content to address disease-specific concerns related to alcohol use within the context of T1D (eg, the effects of alcohol on glucagon efficacy and identification of hypoglycemic symptoms) was developed with input from pediatric endocrinologists, chronic disease epidemiologists, and a developmental behavioral pediatrician specializing in addiction medicine and treating adolescent substance use. Experiential or *slice of life* observations about alcohol use and living with T1D were also incorporated into the intervention using illustrative quotes taken from formative qualitative research [22], with content designed to address the main social and psychological concerns of the target group. Drawings created by an artist embedded in the study team aided in the explanation of both clinical and social-behavioral concepts and were included in the intervention.

Before testing, the intervention underwent formative evaluation through iterative rounds of piloting (for accessibility and acceptability) with patients representing the target group (ie, adolescents and young adults with T1D presenting for routine clinical care to an outpatient hospital clinic). Feedback on the intervention was collected in a structured session with trained members of the research team. The formative evaluation also included a review by pediatric endocrinologists to ensure the accuracy of clinical and health-related content before moving to video production and with stakeholders representing the advocacy organizations through which the study samples were to be recruited. The visual intervention content was then overlaid with the corresponding audio explanation, narrated by either a peer or provider narrator. The narration scripts were identical for both the peer and provider versions of the intervention, with the exception of the initial narrator introductions containing narrator-specific identification (ie, the narrator’s name and role or credentials). The peer and provider narrators were purposefully selected to be of the same gender and same race, and they were similarly styled to reduce the influence on audience perceptions related to factors other than the narrator’s role. Other than the narrator, all video content was identical. The final versions of the intervention (approximately 7.5 minutes in duration) were uploaded to a private channel on a video hosting site and embedded within the REDCap survey so that the participants could automatically view the video without having to exit the survey.

The participants completed a brief survey immediately after viewing the video, and 2 weeks after completion of the *initial* session (baseline survey, intervention video, and immediate postintervention survey), they were sent a follow-up survey through email (automated through REDCap, with up to two additional reminders). The participants received a US $20 gift card for completion of each of the two sessions (initial and follow-up). Of the 138 valid responses at baseline, 134 (97.1%) completed the immediate postintervention survey, and 122 (88.4%) subsequently completed the follow-up assessment. Compared with the participants with complete follow-up, those lost to follow-up were older at diagnosis (13.3 years vs 10.6 years; *P*=.04; data not shown); no other significant differences were observed.

### Covariates

The participants provided sociodemographic data (eg, age, sex, race or ethnicity, parental education, health insurance, enrollment status, school year, living arrangement, and region of college or university attended) and clinical information (eg, age during diagnosis or disease duration, last glycated hemoglobin [HbA_1c_] value, current insulin pump use, current continuous glucose monitoring [CGM] device use, average blood glucose tests per day, average HbA_1c_ tests per year, and self-rated health) at baseline.

### Self-reported Alcohol Use (Primary Outcome)

The participants self-reported alcohol use behaviors at baseline and follow-up assessments, including validated measures of binge use during the past 2 weeks (defined as having 3, 4, 5, or more drinks containing alcohol on one occasion, depending on age and sex threshold [39]). The participants identified any recent events or breaks that were atypical for their schedule in response to novel questions. The events were grouped into those potentially associated with a greater likelihood of drinking (eg, spring break spent with friends) or those with a reduced likelihood of drinking (eg, midterms or final examinations).

### Knowledge, Attitudes, and Concerns Toward Alcohol Use (Secondary Outcomes)

The secondary outcomes relied on novel test batteries that were developed for a series of related studies and informed by clinical reviews and epidemiologic insights [6,22,40,41]. During all three assessments, the participants were asked about their knowledge of the potential impacts of alcohol use on diabetes (10 items with responses of “true,” “false,” or “don’t know”), attitudes toward drinking with diabetes (six items rated from “strongly disagree” [score=1] to “strongly agree” [score=10], with two items only asked among drinkers), and concerns about the potential impact of alcohol on diabetes (seven items rated from “not concerned” [score=0] to “very concerned” [score=6]). To summarize knowledge, the number of correct items was determined. To summarize attitudes and concerns, the mean score across all items was determined for each; higher scores reflected health-protecting attitudes toward drinking or greater concern about the impact of alcohol. Although the measures were novel and not previously validated, they demonstrated reasonable internal consistency (Cronbach α=.9079 for concerns and .6462 for attitudes).

### Intentions and Impressions (Secondary Outcomes)

At postintervention and follow-up assessments, the participants were asked about their impressions of the video (13 items rated from “strongly disagree” [score=1] to “strongly agree” [score=10]) and their intentions (10 items rated from “definitely will not” [score=1] to “definitely will” [score=8], with two items only asked among drinkers). To summarize impressions and intentions, the mean score across all items was determined for each; higher scores reflected more favorable impressions of the intervention (ie, acceptability) or greater intention to engage in lower-risk behaviors. The measures demonstrated reasonable internal consistency (Cronbach α=.8581 for impressions and .8294 for intentions). For aligning with intentions, the participants were asked at the final follow-up to specify actual behaviors, including (1) if they had talked in the past 2 weeks with friends, health care providers, or someone else about how alcohol affects diabetes and (2) the frequency with which they tested their blood sugar before, during, or after drinking during the past 2 weeks.

The participants were also prompted with open-response questions on their diabetes care or alcohol use, first impressions of the video (after the intervention), and final thoughts about the video (final follow-up). The participants who endorsed learning something new or having unanswered questions were prompted for further clarification. These open-ended questions were intended to complement the quantitative impression measures by providing a more subjective and nuanced perspective on intervention acceptability.

### Analytic Approach

**Figure 2:**
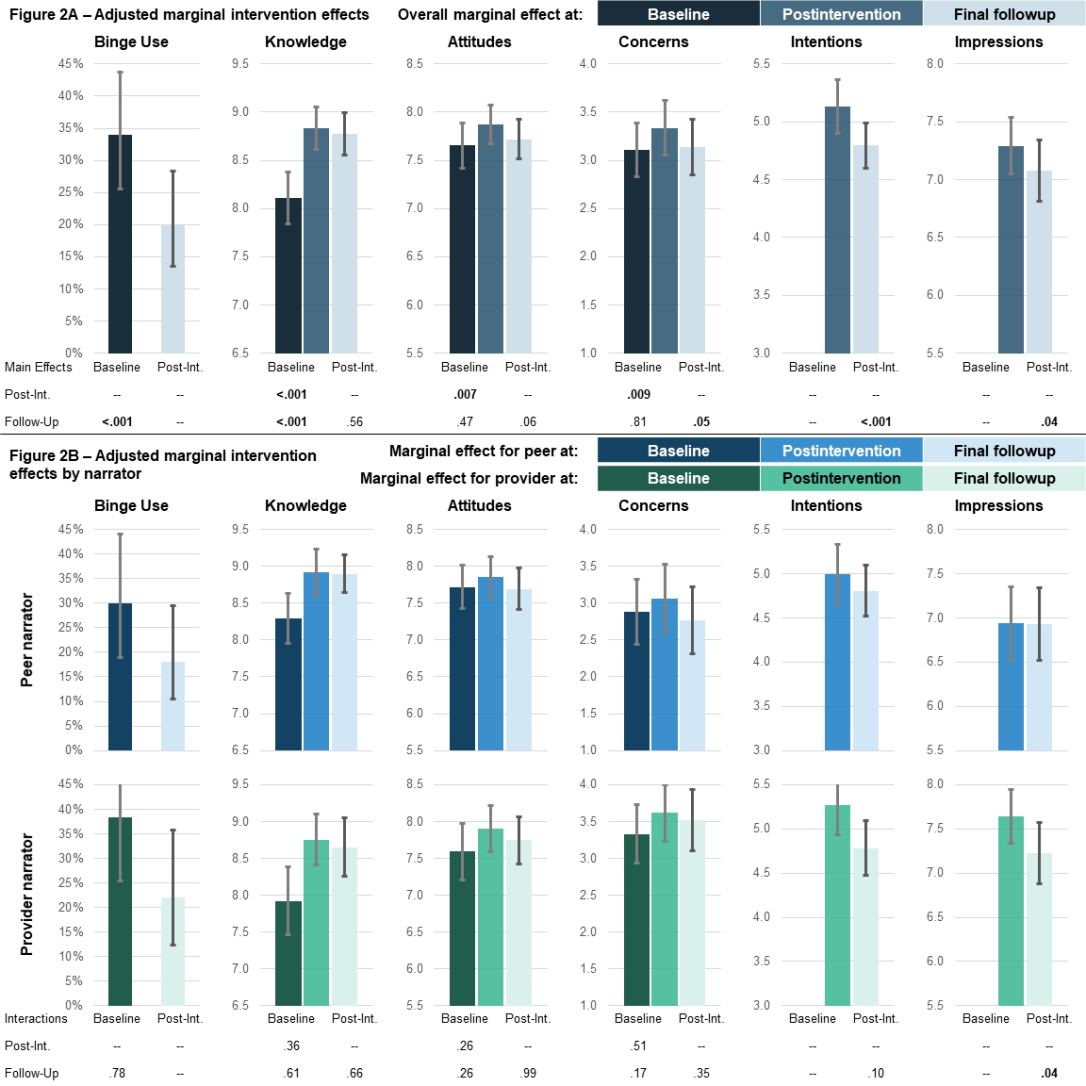
Adjusted marginal intervention effects (y axis) both overall (A, main effects models) and by treatment arm (B, interaction models) across time points (x axis). Adjusted marginal means or probability and 95% CIs were output from multivariate mixed effects models as described in the Methods section. In panel A, the *P* values indicate the statistical significance for the overall intervention effect across time; for example, the intervention reduced the prevalence of binge drinking at final follow-up compared with baseline at *P*<.001. In panel B, the *P* values indicate the statistical significance for the difference in the intervention effect across time by arm (interaction); for example, the reduction in the prevalence of binge drinking at final follow-up compared with baseline was not significantly different by treatment arm at *P*=.78.

The analyses followed a mixed methods protocol, investigating structured survey data and thematically coding optional comment data. Quantitative analyses were performed using SAS version 9.4 (SAS Institute Inc). Statistical significance was set at *P*<.05. All individuals who were randomized into the study and completed the follow-up were included in the analyses (N=122). Differences in characteristics between the arms were assessed using appropriate bivariate tests (Table 1). Differences in outcomes between the arms at baseline and follow-up were evaluated using bivariate tests (Table 2). Multivariate mixed effects models estimated the intervention effects on the outcomes overall (main effects models) and by arm (interaction models; Table 3); adjusted predictive margins were output from these models to visualize the intervention effects (Figure 2). Adjusted regression analysis models controlled for age at survey, age at diagnosis, sex, race or ethnicity, parent education, last HbA_1c_ value, and CGM device use, while accounting for repeated measures; the models for alcohol use outcomes were additionally adjusted for atypical events affecting the likelihood of drinking. We selected measures for covariate adjustment based on their known association with alcohol use behaviors (eg, age and sex), known association with diabetes knowledge (eg, HbA_1c_ value), or imbalance across treatment arm (eg, parental education). We additionally evaluated the secondary outcomes (change in knowledge, attitudes, and concerns from baseline to after the intervention) as predictors of the overall change in binge drinking (at final follow-up vs baseline). The participants’ responses to the open-response questions were analyzed using an iterative process, including open coding, to identify emergent themes [22] and refinement of the coding scheme over several rounds of joint review with 3 coders (RKT, LEW, and ERW). Disagreements were resolved through discussion and consensus. Themes that reflected consistent commonality across the responses or important distinctive motifs were specified; illustrative quotes for each theme were selected and summarized as context to understand the impacts of the intervention (Multimedia Appendix 1). All 122 participants provided a textual response for their impression of the video, and 91 provided a textual response for the remaining optional prompts; the response rates did not differ by arm (*P*=.40).

**Table 1:** Sociodemographic and clinical characteristics by intervention narrator (N=122).

Characteristics			Total	Peer	Provider	*P* value	
Total, n (%)			122 (100)	63 (51.6)	59 (48.4)	N/A^a^	
**Sociodemographic characteristics**							
	**Age at survey (years), mean (SD)**		20.46 (1.44)	20.35 (1.48)	20.58 (1.40)	.35	
	**Sex, n (%)**						.86
		Male	24 (19.7)	12 (19)	12 (20.3)		
		Female	98 (80.3)	51 (81)	47 (79.7)		
	**Race and ethnicity, n (%)**						.25
		White or non-Hispanic	102 (83.6)	55 (87.3)	47 (79.7)		
		Person of color or Hispanic	20 (16.4)	8 (12.7)	12 (20.3)		
	**Parental education, n (%)**						.02
		High school degree or less	8 (6.6)	2 (3.2)	6 (10.2)		
		Some college, no degree	16 (13.1)	6 (9.5)	10 (16.9)		
		Associate’s degree	13 (10.7)	7 (11.1)	6 (10.2)		
		Bachelor’s degree	43 (35.2)	18 (28.6)	25 (42.4)		
		Graduate degree	41 (33.6)	30 (47.6)	11 (18.6)		
		Unsure	1 (0.8)	0 (0)	1 (1.7)		
	**Health insurance, n (%)**						.78
		Parent’s plan	108 (88.5)	57 (90.5)	51 (86.4)		
		Own plan (including school plan)	7 (5.7)	3 (4.8)	4 (6.8)		
		Public or uninsured	7 (5.7)	3 (4.8)	4 (6.8)		
	**Enrollment status, n (%)**						.68
		Full time	109 (89.3)	57 (90.5)	52 (88.1)		
		Part time or other	13 (10.7)	6 (9.5)	7 (11.9)		
	**Year in school, n (%)**						.94
		Freshman	14 (11.5)	8 (12.7)	6 (10.2)		
		Sophomore	38 (31.1)	20 (31.7)	18 (30.5)		
		Junior	38 (31.1)	19 (30.2)	19 (32.2)		
		Senior	20 (16.4)	9 (14.3)	11 (18.6)		
		Fifth year or graduate student	12 (9.8)	7 (11.1)	5 (8.5)		
	**Living arrangement during school, n (%)**						.18
		At home, with parent, or guardian	23 (18.9)	9 (14.3)	14 (23.7)		
		On or off campus housing	99 (81.1)	54 (85.7)	45 (76.3)		
	**Region of college or university, n (%)**						0.28
		Northeast	34 (27.9)	17 (27)	17 (28.8)		
		Midwest	29 (23.8)	14 (22.2)	15 (25.4)		
		South	46 (37.7)	27 (42.9)	19 (32.2)		
		West	9 (7.4)	2 (3.2)	7 (11.9)		
		Outside the United States	4 (3.3)	3 (4.8)	1 (1.7)		
**Clinical characteristics**							
	Age at diagnosis (years), mean (SD)		10.56 (5.14)	9.89 (5.28)	11.27 (4.93)	.08	
	Disease duration (years), mean (SD)		9.90 (5.32)	10.46 (5.48)	9.31 (5.12)	.18	
	Last HbA_1c_^b^ value, mean (SD)		7.65 (1.20)	7.47 (1.10)	7.84 (1.27)	.06	
	**Insulin pump use, n (%)**						.69
		Not using	21 (17.2)	10 (15.9)	11 (18.6)		
		Currently using	101 (82.8)	53 (84.1)	48 (81.4)		
	**Continuous glucose monitor use, n (%)**						.08
		Not using	48 (39.3)	20 (31.7)	28 (47.5)		
		Currently using	74 (60.7)	43 (68.3)	31 (52.5)		
	**Average blood glucose tests (times per day), n (%)**						.22
		0-2	25 (20.5)	16 (25.4)	9 (15.3)		
		3-4	45 (36.9)	25 (39.7)	20 (33.9)		
		5-6	32 (26.2)	12 (19)	20 (33.9)		
		≥7	20 (16.4)	10 (15.9)	10 (16.9)		
	**Average HbA_1c_ tests per year, n (%)**						.06
		0-1	12 (9.8)	5 (7.9)	7 (11.9)		
		2	31 (25.4)	19 (30.2)	12 (20.3)		
		3	36 (29.5)	23 (36.5)	13 (22)		
		≥4	43 (35.2)	16 (25.4)	27 (45.8)		
	**Self-rated health, n (%)**						.77
		Fair or poor	13 (10.7)	5 (7.9)	8 (13.6)		
		Good	56 (45.9)	29 (46)	27 (45.8)		
		Very good	46 (37.7)	25 (39.7)	21 (35.6)		
		Excellent	7 (5.7)	4 (6.3)	3 (5.1)		
	**Past-year alcohol use, n (%)**						.94
		None	21 (17.2)	11 (17.5)	10 (16.9)		
		Any	101 (82.8)	52 (82.5)	49 (83.1)		

^a^N/A: not applicable.

^b^HbA_1c_: glycated hemoglobin.

**Table 2:** Unadjusted outcomes at baseline and follow-up by narrator (N=122)^a^.

Characteristics			Total		Peer		Provider		*P* value
**Past 2-week binge use (self-reported), n (%)**									
	Baseline (any)	43 (35.2)		21 (33.3)		22 (37.3)		.65	
	Follow-up (any)	26 (21.3)		14 (22.2)		12 (20.3)		.80	
	Difference at follow-up versus baseline	–13.9		–11.1		–16.9		.48	
**Knowledge summary^b^**									
	Baseline, mean (SD)	8.11 (1.58)		8.35 (1.30)		7.86 (1.81)		.20	
	Postintervention, mean (SD)	8.84 (1.28)		8.97 (1.15)		8.69 (1.41)		.27	
	Follow-up, mean (SD)	8.78 (1.29)		8.95 (0.92)		8.59 (1.58)		.32	
	Difference at postintervention versus baseline	+0.72		+0.62		+0.83		.75	
	Difference at follow-up versus baseline	+0.66		+0.60		+0.73		.97	
**Attitude summary^c^**									
	Baseline, mean (SD)	7.66 (1.38)		7.71 (1.27)		7.61 (1.50)		.81	
	Postintervention, mean (SD)	7.87 (1.25)		7.84 (1.21)		7.91 (1.29)		.63	
	Follow-up, mean (SD)	7.72 (1.24)		7.68 (1.24)		7.76 (1.24)		.77	
	Difference at postintervention versus baseline	+0.22		+0.13		+0.31		.20	
	Difference at follow-up versus baseline	+0.06		–0.03		+0.15		.44	
**Concern summary^d^**									
	Baseline, mean (SD)	3.10 (1.62)		2.92 (1.67)		3.29 (1.55)		.23	
	Postintervention, mean (SD)	3.33 (1.67)		3.09 (1.76)		3.58 (1.55)		.15	
	Follow-up, mean (SD)	3.13 (1.70)		2.80 (1.72)		3.48 (1.62)		.02	
	Difference at postintervention versus baseline	+0.23		+0.17		+0.29		.84	
	Difference at follow-up versus baseline	+0.03		–0.12		+0.19		.14	
**Intention summary^e^**									
	Postintervention, mean (SD)	5.13 (1.39)		4.94 (1.43)		5.33 (1.33)		.10	
	Follow-up, mean (SD)	4.80 (1.18)		4.76 (1.25)		4.84 (1.10)		.67	
	Difference at follow-up versus postintervention	–0.33		–0.18		–0.49		.08	
**Impression summary^f^**									
	Postintervention, mean (SD)	7.28 (1.48)		6.95 (1.67)		7.64 (1.14)		.03	
	Follow-up, mean (SD)	7.07 (1.53)		6.94 (1.68)		7.22 (1.35)		.38	
	Difference at follow-up versus postintervention	–0.21		–0.01		–0.42		.07	
**In past 2 weeks...(at follow-up), n (%)**									
	Talked with anyone about alcohol and type 1 diabetes	68 (55.7)		39 (61.9)		29 (49.2)		.16	
	Always tested before, during, or after drinking	54 (44.3)		23 (36.5)		31 (52.5)		.07	

^a^*P* values compare the peer versus provider arms.

^b^The knowledge summary indicates the number of correct items out of a total of 10 items.

^c^The attitude summary reflects the mean (SD) score across six items for drinkers and four items for nondrinkers; the response options ranged from “strongly disagree” (score=1) to “strongly agree” (score=10).

^d^The concern summary reflects the mean (SD) score across seven items; the response options ranged from “not concerned” (score=0) to “very concerned” (score=6).

^e^The intention summary reflects the mean (SD) score across 10 items for drinkers and eight items for nondrinkers; the response options ranged from “definitely will not” (score=1) to “definitely will” (score=8).

^f^The impression summary reflects the mean (SD) score across 12 items; the response options ranged from “strongly disagree” (score=1) to “strongly agree” (score=10).

**Table 3:** Adjusted regression analysis results^a^.

Characteristics		Main effects models			Interaction models	
		Estimate (95% CI)	*P* value	Estimate (95% CI)		*P* value
**Past 2-week binge^b^**						
	Peer versus provider	0.72 (0.33 to 1.60)	.42	0.69 (0.29 to 1.67)		.41
	Follow-up versus baseline	0.48 (0.31 to 0.75)	<.001	0.45 (0.25 to 0.83)		.01
	Peer×follow-up	—^c^	—	1.14 (0.46 to 2.79)		.78
**Knowledge^d^**						
	Peer versus provider	0.25 (–0.21 to 0.72)	.28	0.37 (–0.22 to 0.96)		.22
	Postintervention versus baseline	0.72 (0.50 to 0.95)	<.001	0.83 (0.46 to 1.20)		<.001
	Follow-up versus baseline	0.66 (0.42 to 0.91)	<.001	0.73 (0.32 to 1.14)		<.001
	Peer×postintervention	—	—	–0.21 (–0.67 to 0.24)		.36
	Peer×follow-up	—	—	–0.13 (–0.61 to 0.36)		.61
**Attitudes^e^**						
	Peer versus provider	–0.08 (–0.48 to 0.33)	.71	0.13 (–0.37 to 0.63)		.62
	Postintervention versus baseline	0.22 (0.06 to 0.37)	.007	0.31 (0.06 to 0.56)		.02
	Follow-up versus baseline	0.06 (–0.10 to 0.22)	.47	0.15 (–0.08 to 0.38)		.20
	Peer×postintervention	—	—	–0.18 (–0.49 to 0.13)		.26
	Peer×follow-up	—	—	–0.18 (–0.50 to 0.13)		.26
**Concerns^f^**						
	Peer versus provider	–0.59 (–1.19 to 0.01)	.05	–0.44 (–1.06 to 0.18)		.16
	Postintervention versus baseline	0.23 (0.06 to 0.40)	.009	0.29 (–0.01 to 0.58)		.06
	Follow-up versus baseline	0.03 (–0.19 to 0.25)	.81	0.19 (–0.15 to 0.52)		.28
	Peer×postintervention	—	—	–0.12 (–0.46 to 0.23)		.51
	Peer×follow-up	—	—	–0.31 (–0.75 to 0.13)		.17
**Intentions^g^**						
	Peer versus provider	–0.13 (–0.57 to 0.31)	.56	–0.28 (–0.78 to 0.22)		.27
	Follow-up versus postintervention	–0.33 (–0.51 to –0.15)	<.001	–0.49 (–0.78 to –0.20)		.01
	Peer×follow-up	—	—	0.30 (–0.06 to 0.67)		.10
**Impressions^h^**						
	Peer versus provider	–0.50 (–1.01 to 0.01)	.05	–0.71 (–1.25 to –0.17)		.01
	Follow-up versus postintervention	–0.21 (–0.40 to –0.01)	.04	–0.42 (–0.74 to –0.10)		.01
	Peer×follow-up	—	—	0.41 (0.02 to 0.80)		.04

^a^All models were adjusted for age at survey, age at diagnosis, sex, race or ethnicity, parent education, last glycated hemoglobin value, and continuous glucose monitoring device use. Models for binge use additionally adjust for atypical events that affect the likelihood of drinking. The *peertime interaction* coefficient represents the difference in the change over time for the peer arm versus the provider arm (only included in *Interaction* models); this indicates if the effect of the intervention differed by arm. When not statistically significant at *P*<.05, results from the *Main Effects* column are preferred.

^b^Self-reported past 2-week binge alcohol use was modeled with a binomial distribution. Odds ratios are shown instead of β coefficients.

^c^These are models in which this term was not included.

^d^The knowledge summary score indicates the number of correct items out of a total of 10 items, modeled with a normal distribution.

^e^The attitude summary score (average of six items for drinkers and four items for nondrinkers, scored from “strongly disagree” [score=1] to “strongly agree” [score=10]) was modeled with a normal distribution.

^f^The concern summary score (average of seven items, scored from “not concerned” [score=0] to “very concerned” [score=6]) was modeled with a normal distribution.

^g^The intention summary score (average of 10 items for drinkers and eight items for nondrinkers, scored from “definitely will not” [score=1] to “definitely will” [score=8]) was modeled with a normal distribution.

^h^The impression summary score (average of 12 items, scored from “strongly disagree” [score=1] to “strongly agree” [score=10]) was modeled with a normal distribution.

## Results

### Sample Characteristics

The participants were aged 20.46 (SD 1.44) years; 80.3% (98/122) were women, 83.6% (102/122) were White and non-Hispanic, 68.9% (84/122) had a parent with a bachelor’s degree or higher, 82.8% (101/122) used insulin pumps, 60.7% (74/122) used CGM devices, their last HbA_1c_ value was 7.65 (SD 1.20), and 82.8% (101/122) self-reported past-year alcohol use (Table 1). The characteristics were largely balanced across the peer (63/122, 51.6%) and provider (59/122, 48.4%) arms, except for the fact that the peer arm participants reported higher parental education (*P*=.02) than their counterparts.

### Knowledge, Attitudes, and Concerns

Despite the participants’ high baseline knowledge (participants averaged 8.11, SD 1.58 correct items out of 10; Table 2), there was a significant adjusted improvement in correct knowledge items of +0.72 after the intervention (95% CI 0.50-0.95; *P*<.001; Table 3; Figure 2) and +0.66 at follow-up (95% CI 0.42-0.91; *P*<.001); the improvements after the intervention and at follow-up were not significantly different by arm (*P*=.36 and *P*=.61, respectively). The improvements were driven by an increased understanding of the impact of alcohol on the liver, the distinction between being drunk and low blood glucose, the interaction between alcohol and glucagon, and alcohol-related dehydration (Multimedia Appendix 2).

The participants saw adjusted improvements in health-protecting attitudes of +0.22 after the intervention compared with baseline (95% CI 0.06-0.37; *P*=.007; Table 3; Figure 2); overall improvements after the intervention were similar by arm (*P*=.26). Attitudes did not differ between the follow-up and baseline (+0.06, 95% CI –0.10 to 0.22; *P*=.47). Postintervention changes were driven by attitudes related to telling friends that they cannot drink because of diabetes, and at follow-up, fewer college students agreed that college students with diabetes could drink if they are careful (Multimedia Appendix 2).

The participants reported adjusted increases in concerns about alcohol of +0.23 after the intervention versus baseline (95% CI 0.06-0.40; *P*=.009; Table 3; Figure 2); improvements after the intervention were similar by arm (*P*=.51). Concerns were not different at follow-up versus baseline (+0.03, 95% CI –0.19 to 0.25; *P*=.81). Postintervention changes were driven by increased concern for the effectiveness of glucagon while drinking, whereas increased concerns about the impact of alcohol on HbA_1c_ tests were greater for those in the provider arm than for those in the peer arm (Multimedia Appendix 2).

### Intentions and Impressions

Adjusted overall intentions to engage in lower-risk behaviors decreased from after the intervention to final follow-up (–0.33, 95% CI –0.51 to –0.15; *P*<.001; Table 3). Positive impressions of the intervention decreased for those in the provider arm (–0.42, 95% CI –0.74 to –0.10; *P*=.01), whereas those in the peer arm had lower impressions than those in the provider arm (–0.71, 95% CI –1.25 to –0.17; *P*=.01), but their impressions were stable over time (interaction +0.41, 95% CI 0.02-0.80; *P*=.04). The adjusted ratings of narrator knowledge and trust were significantly higher for the provider arm than for the peer arm (Multimedia Appendix 2). After the intervention, the provider arm participants also rated their trust in the information presented and willingness to share this video more highly than the peer arm participants. The provider arm was significantly more likely to decrease their rating of whether they learned something new from after the intervention to follow-up than the peer arm. Intentions to tell their friends about the effect of alcohol on diabetes and to ask their provider about diabetes and alcohol use were stable for the peer arm but more likely to decrease at follow-up for the provider arm.

### Alcohol Use Outcomes

Overall, the intervention was associated with a 52% adjusted relative reduction in self-reported binge drinking (odds ratio [OR] 0.48, 95% CI 0.31-0.75; *P*=.001; Table 3; Figure 2) from baseline to follow-up, with no significant difference across narrators (*P*=.78). The change in concerns from baseline to after the intervention was significantly associated with a reduction in binge drinking at follow-up (OR 0.71, 95% CI 0.58-0.88; data not shown), whereas the changes in knowledge and attitudes were not (knowledge OR 0.93, 95% CI 0.75-1.15; attitudes OR 0.90, 95% CI 0.70-1.15).

### Qualitative Outcomes

Overall, the qualitative responses suggested that the participants seemed to derive value from the intervention, with four primary themes emerging (Multimedia Appendix 1). First, the youth want disease-specific information about alcohol and have various sources of knowledge acquisition, including through the intervention. Some participants were disappointed that they had not previously been exposed to information about the impact of alcohol on diabetes from other sources (such as their care providers), whereas others expressed appreciation for receiving this new information during the study. However, the information was not novel to all participants, and some described prior acquisition of knowledge through sources such as the internet or peers, as well as their health care teams. Second, the youth noted the influence of personal safety on alcohol use decision-making and the desire for harm reduction messages, as well as new safety practices motivated by the intervention. The participants recognized the prevalence of alcohol use among college students with T1D, expressing a preference for video content encouraging safe and responsible use rather than complete abstinence. Some participants commented upon current safety practices such as drinking with friends and checking their blood sugar while consuming alcohol, whereas others discussed new behaviors inspired by the video, such as recognition of their need to monitor and adjust for their own alcohol intake limits. The third theme encompasses the appeal of intervention content that is not only developmentally appropriate and affirming of personal experiences but also contains an authoritative voice and is professionally produced. Many participants remarked upon their ability to relate to the video because of the relevance of the information and trustworthiness of the speakers, whereas others yearned for more personal stories, easily digestible content, and improved production quality. Finally, the youth addressed factors complicating diabetes and alcohol use management at college, including the need for and role of adequate systems of support. The participants elucidated ongoing challenges faced by youth with T1D, such as social isolation from peers without T1D, inadequate access to specialists on campus, and difficulty balancing their health with other competing priorities during the college years.

## Discussion

### Principal Findings

This pilot trial demonstrates that a novel, web-based psychoeducational intervention reduces self-reported binge drinking 2 weeks after the intervention among college students with T1D, irrespective of the narrator. Both narrators improved knowledge of the effects of alcohol and temporarily affected attitudes toward and concerns about drinking, although the provider narrator was more highly rated regarding subject knowledge and trustworthiness. Given measurable reductions in binge drinking at follow-up, this brief video intervention demonstrates promise for mitigating risky alcohol consumption among college students with T1D, and the web-based format is suitable for delivery across numerous settings.

The substantial reduction in binge alcohol use observed for the participants in both arms suggests that both peer and provider narrators can motivate behavioral change. However, the mechanisms by which the narrator affects change may be different. The provider narrator appeared to quantitatively *win out* over the peer narrator with respect to perceived knowledge and trustworthiness, suggesting that delivery of factual content may be enhanced by the aura of clinical authority or that some youths strongly trust and respond to perceived medical experts. Prior qualitative work reported that youths living with chronic conditions conveyed an emphatic preference for the inclusion of information about alcohol use in subspecialty care and reported thinking that their specialty care team know them and their disease best [22]; current qualitative findings echo this and indicate high respect for providers’ authority on this topic. The peer narrator appeared to better motivate the intention of the participants to speak with others about the effects of alcohol on their diabetes, suggesting that emotional processing and sharing of experiences may be important for motivating behavioral change and reinforcing knowledge gains. The qualitative findings also supported the participants’ desire for narrator and content relatability, as the participants responded positively to hearing their own experiences reflected by their peers. Subtle differences in secondary outcomes by narrator underscore the need for purposeful, well-designed future work to tease out the components that produce cognitive, affective, and behavioral changes, including over a longer time horizon, using insights from pilot work to design and refine a robust intervention suitable for testing in a traditional, controlled trial, consistent with best practice recommendations for behavioral trials [42].

Future work should evaluate delivery on a longer timescale and determine if greater personalization might solidify and sustain the effects to extend the promising results from this pilot. As changes in concerns about alcohol and diabetes (an intermediate outcome predicting a reduction in binge use) were not sustained at the final follow-up, the single-dose intervention appeared to alert the participants to possible dangers, generating a near-term response of increased concern. The lack of sustained change may reflect recalibration of concern, given time to absorb new information (knowledge and experience), growth in perceived self-efficacy for navigating drinking, or retrenchment from the initial state of alarm. A model that delivers multiple doses (boosters) may help to reinforce and sustain the response; hence, future studies should investigate changes over longer periods for a single and possibly multiple-dose intervention. Both narrators in this trial were unknown to the participants; intervention delivery from someone with whom participants have an established relationship, such as a trusted provider [43] or close friend, may also boost intervention effects; however, these designs would limit the scalability of a broadly diffusible digital intervention. Alternatively, the effectiveness of a one-time interaction may be enhanced by providing youths with the opportunity to subsequently discuss the intervention’s educational content with a trusted provider or peer.

Although in-person anticipatory conversations between adolescents or young adults and their diabetes care teams about the effects of alcohol on their health and strategies for avoiding risk may be the gold standard for delivering a preventive intervention, this model may not be practical. Providers are increasingly tasked with covering a myriad of topics during short clinical encounters [44], and health guidance is increasingly delivered through web-based channels. Therefore, understanding the acceptability and effects of electronic interventions is vital [45]. Prior work has demonstrated that web-based information and diabetes support can augment clinical care [46] and improve quality of life [47]. This study demonstrates that digitally delivered interventions can effectively engage college students with T1D and provide them with vital health information, a promising model for larger-scale dissemination [25,48]. Future research and implementation studies are needed to evaluate the long-term effectiveness of digital tools and build evidence around best practices for content and delivery [49,50].

Finally, many of the participants noted that they would have liked to receive this intervention earlier (ie, before they began drinking or matriculated to college). As the intervention materials were not specific only to college, the current intervention might be generalizable for use with younger individuals or young adults not attending college, and such extensions could be tested. However, as this intervention was designed to focus on harm reduction, further investigation, including with respect to timing of or age at delivery, is needed to determine if these materials are suitable for the goal of preventing drinking initiation.

### Limitations

This study includes several limitations. First, as with all internet-based recruitment, the underlying population (*denominator*) [51] is unclear, making it difficult to characterize sample representativeness and imposing constraints on generalizability. Although a prior methodological evaluation identified that this approach yields a sample that is representative of the sampling frame [35], this sample likely does not represent all college students with T1D (eg, this cohort included predominantly women, and most of the participants were privately insured, had higher pump and CGM use, and also had better self-reported glycemic control than other similarly aged cohorts [52]). This sample may be socioeconomically advantaged, with well-managed diabetes; hence, the findings may be indicative of a *best case scenario*. Second, this study was designed as a pilot trial to gauge the acceptability and relative impacts of the narrator and lacked a nonintervention control group. The observed effects could stem from test-retest bias, carryover effects, or regression to the mean; future controlled evaluations might mitigate these concerns. Third, all outcomes (and diabetes status) were self-reported, and only the alcohol use questions relied on validated items. To our knowledge, there are no pre-existing validated tools to assess knowledge, attitudes, and concerns regarding the intersection of alcohol use and diabetes. The survey items were extensively pretested and have been used in other surveys [6,37,38]; however, they may be subject to measurement errors and other biases stemming from self-reporting. Despite the use of validated items for alcohol use, self-reported behaviors are subject to social desirability bias, which may result in misclassification. Finally, although the intervention videos were made to be maximally comparable (eg, same-gendered, same-race, and similarly styled narrators), other differences may have existed that differentially affected the response to the intervention, including among certain participant subgroups (eg, men respond differently to female narrators). Relying on an actor or more professional video editing, qualitatively noted by some participants, may diminish the potential differences.

### Conclusions

We demonstrate the acceptability and impacts of a psychoeducational intervention for mitigating binge alcohol use among college students with T1D. We further determine that peer and provider narrators have similar short-term impacts as framing devices for this educational content. As this pilot demonstrates the feasibility of delivering this intervention to a hard-to-reach group through web-based channels, similar outreach methods could be used to deliver this content more broadly. In light of these promising findings, future work should further test these materials against the control of *care as usual* and should determine what further modifications are needed to enhance intervention effects.

## Appendix

Multimedia Appendix 1 Qualitative themes regarding intervention impressions and impact.

Multimedia Appendix 2 Adjusted results for specific items.

## Abbreviations

CGM: continuous glucose monitoring
CONSORT: Consolidated Standards of Reporting Trials
HbA_1c_: glycated hemoglobin
OR: odds ratio
REDCap: Research Electronic Data Capture
T1D: type 1 diabetes

## References

[1] Modi AC, Pai AL, Hommel KA, Hood KK, Cortina S, Hilliard ME, Guilfoyle SM, Gray WN, Drotar D (2012). Pediatric self-management: a framework for research, practice, and policy. Pediatrics.

[2] McCarthy MM, Grey M (2018). Type 1 diabetes self-management from emerging adulthood through older adulthood. Diabetes Care.

[3] Barnard K, Sinclair JM, Lawton J, Young AJ, Holt RI (2012). Alcohol-associated risks for young adults with Type 1 diabetes: a narrative review. Diabet Med.

[4] Jaser SS, Yates H, Dumser S, Whittemore R (2011). Risky business: risk behaviors in adolescents with type 1 diabetes. Diabetes Educ.

[5] Hanna KM, Woodward J (2013). The transition from pediatric to adult diabetes care services. Clin Nurse Spec.

[6] Weitzman ER, Ziemnik RE, Huang Q, Levy S (2015). Alcohol and marijuana use and treatment nonadherence among medically vulnerable youth. Pediatrics.

[7] Lemly DC, Lawlor K, Scherer EA, Kelemen S, Weitzman ER (2014). College health service capacity to support youth with chronic medical conditions. Pediatrics.

[8] Hanna KM, Stupiansky NW, Weaver MT, Slaven JE, Stump TE (2014). Alcohol use trajectories after high school graduation among emerging adults with type 1 diabetes. J Adolesc Health.

[9] Wisk LE, Weitzman ER (2016). Substance use patterns through early adulthood: results for youth with and without chronic conditions. Am J Prev Med.

[10] Knight JR, Wechsler H, Kuo M, Seibring M, Weitzman ER, Schuckit MA (2002). Alcohol abuse and dependence among U.S. college students. J Stud Alcohol.

[11] Hingson RW, Zha W, Weitzman ER (2009). Magnitude of and trends in alcohol-related mortality and morbidity among U.S. college students ages 18-24, 1998-2005. J Stud Alcohol Drugs Suppl.

[12] Weitzman ER, Nelson TF, Wechsler H (2003). Taking up binge drinking in college: the influences of person, social group, and environment. J Adolesc Health.

[13] Harjutsalo V, Feodoroff M, Forsblom C, Groop P, FinnDiane Study Group (2014). Patients with Type 1 diabetes consuming alcoholic spirits have an increased risk of microvascular complications. Diabet Med.

[14] MacNaught N, Holt P (2015). Type 1 diabetes and alcohol consumption. Nurs Stand.

[15] Barnard KD, Dyson P, Sinclair JM, Lawton J, Anthony D, Cranston M, Holt RI (2014). Alcohol health literacy in young adults with type 1 diabetes and its impact on diabetes management. Diabet Med.

[16] Tamony P, Holt R, Barnard K (2015). The role of mobile applications in improving alcohol health literacy in young adults with type 1 diabetes: help or hindrance?. J Diabetes Sci Technol.

[17] Knychala MA, Jorge ML, Muniz CK, Faria PN, Jorge PT (2015). High-risk alcohol use and anxiety and depression symptoms in adolescents and adults with type 1 diabetes mellitus: a cross-sectional study. Diabetol Metab Syndr.

[18] Ramsey SE, Engler PA, Harrington M, Smith RJ, Fagan MJ, Stein MD, Friedmann P (2010). Brief alcohol intervention among at-risk drinkers with diabetes. Subst Abuse.

[19] Lakey WC, Barnard K, Batch BC, Chiswell K, Tasneem A, Green JB (2013). Are current clinical trials in diabetes addressing important issues in diabetes care?. Diabetologia.

[20] Engler PA, Ramsey SE, Smith RJ (2013). Alcohol use of diabetes patients: the need for assessment and intervention. Acta Diabetol.

[21] Powell PW, Corathers SD, Raymond J, Streisand R (2015). New approaches to providing individualized diabetes care in the 21st century. Curr Diabetes Rev.

[22] Weitzman ER, Salimian PK, Rabinow L, Levy S (2019). Perspectives on substance use among youth with chronic medical conditions and implications for clinical guidance and prevention: A qualitative study. PLoS One.

[23] Spijkerman R, Roek MA, Vermulst A, Lemmers L, Huiberts A, Engels RC (2010). Effectiveness of a web-based brief alcohol intervention and added value of normative feedback in reducing underage drinking: a randomized controlled trial. J Med Internet Res.

[24] Norman P, Cameron D, Epton T, Webb TL, Harris PR, Millings A, Sheeran P (2018). A randomized controlled trial of a brief online intervention to reduce alcohol consumption in new university students: Combining self-affirmation, theory of planned behaviour messages, and implementation intentions. Br J Health Psychol.

[25] Kerr D, King F, Klonoff DC (2019). Digital Health Interventions for Diabetes: Everything to Gain and Nothing to Lose. Diabetes Spectr.

[26] Jones E, Sinclair JM, Holt RI, Barnard KD (2013). Social networking and understanding alcohol-associated risk for people with type 1 diabetes: friend or foe?. Diabetes Technol Ther.

[27] Kistin C, Silverstein M (2015). Pilot Studies: A critical but potentially misused component of interventional research. J Am Med Assoc.

[28] Mohr DC, Schueller SM, Montague E, Burns MN, Rashidi P (2014). The behavioral intervention technology model: an integrated conceptual and technological framework for eHealth and mHealth interventions. J Med Internet Res.

[29] Piette JD, Resnicow K, Choi H, Heisler M (2013). A diabetes peer support intervention that improved glycemic control: mediators and moderators of intervention effectiveness. Chronic Illn.

[30] Heisler M (2010). Different models to mobilize peer support to improve diabetes self-management and clinical outcomes: evidence, logistics, evaluation considerations and needs for future research. Fam Pract.

[31] Brownson CA, Heisler M (2009). The role of peer support in diabetes care and self-management. Patient.

[32] Fisher EB, Boothroyd RI, Elstad EA, Hays L, Henes A, Maslow GR, Velicer C (2017). Peer support of complex health behaviors in prevention and disease management with special reference to diabetes: systematic reviews. Clin Diabetes Endocrinol.

[33] Heisler M, Vijan S, Makki F, Piette JD (2010). Diabetes control with reciprocal peer support versus nurse care management: a randomized trial. Ann Intern Med.

[34] Kaselitz E, Shah M, Choi H, Heisler M (2019). Peer characteristics associated with improved glycemic control in a randomized controlled trial of a reciprocal peer support program for diabetes. Chronic Illn.

[35] Wisk LE, Nelson EB, Magane KM, Weitzman ER (2019). Clinical trial recruitment and retention of college students with type 1 diabetes via social media: an implementation case study. J Diabetes Sci Technol.

[36] Levy S, Dedeoglu F, Gaffin JM, Garvey KC, Harstad E, MacGinnitie A, Rufo PA, Huang Q, Ziemnik RE, Wisk LE, Weitzman ER (2016). A screening tool for assessing alcohol use risk among medically vulnerable youth. PLoS One.

[37] Weitzman ER, Magane KM, Wisk LE, Allario J, Harstad E, Levy S (2018). Alcohol use and alcohol-interactive medications among medically vulnerable youth. Pediatrics.

[38] Wisk LE, Magane KM, Levy S, Weitzman ER (2020). Alcohol use behaviors and reasons to abstain from or limit drinking among medically vulnerable youth. J Addict Med.

[39] (2015). Alcohol screening and brief intervention for youth: A practitioner's guide. National Institute on Alcohol Abuse and Alcoholism, American Academy of Pediatrics.

[40] Johnston L, O'Malley P, Miech R, Bachman J, Schulenberg J, Patrick M (2019). Monitoring the future national survey results on drug use, 1975-2018: overview, key findings on adolescent drug use. Institute for Social Research Report, The University of Michigan, MI.

[41] Mallett KA, Varvil-Weld L, Turrisi R, Read A (2011). An examination of college students' willingness to experience consequences as a unique predictor of alcohol problems. Psychol Addict Behav.

[42] Collins LM, Murphy SA, Strecher V (2007). The multiphase optimization strategy (MOST) and the sequential multiple assignment randomized trial (SMART): new methods for more potent eHealth interventions. Am J Prev Med.

[43] Datye KA, Moore DJ, Russell WE, Jaser SS (2015). A review of adolescent adherence in type 1 diabetes and the untapped potential of diabetes providers to improve outcomes. Curr Diab Rep.

[44] Hilliard ME, De Wit M, Wasserman RM, Butler AM, Evans M, Weissberg-Benchell J, Anderson BJ (2018). Screening and support for emotional burdens of youth with type 1 diabetes: strategies for diabetes care providers. Pediatr Diabetes.

[45] Harris SK, Aalsma MC, Weitzman ER, Garcia-Huidobro D, Wong C, Hadland SE, Santelli J, Park MJ, Ozer EM (2017). Research on clinical preventive services for adolescents and young adults: where are we and where do we need to go?. J Adolesc Health.

[46] Nicholas DB, Fellner KD, Frank M, Small M, Hetherington R, Slater R, Daneman D (2012). Evaluation of an online education and support intervention for adolescents with diabetes. Soc Work Health Care.

[47] Jaser SS, Whittemore R, Chao A, Jeon S, Faulkner MS, Grey M (2014). Mediators of 12-month outcomes of two Internet interventions for youth with type 1 diabetes. J Pediatr Psychol.

[48] Kerr D, Gabbay RA, Klonoff DC (2018). Finding real value from digital diabetes health: Is digital health dead or in need of resuscitation?. J Diabetes Sci Technol.

[49] Klonoff DC (2019). Behavioral theory: the missing ingredient for digital health tools to change behavior and increase adherence. J Diabetes Sci Technol.

[50] Reidy C, Klonoff DC, Barnard-Kelly KD (2019). Supporting good intentions with good evidence: how to increase the benefits of diabetes social media. J Diabetes Sci Technol.

[51] Chunara R, Wisk LE, Weitzman ER (2017). Denominator issues for personally generated data in population health monitoring. Am J Prev Med.

[52] Foster NC, Beck RW, Miller KM, Clements MA, Rickels MR, DiMeglio LA, Maahs DM, Tamborlane WV, Bergenstal R, Smith E, Olson BA, Garg SK (2019). State of type 1 diabetes management and outcomes from the T1D exchange in 2016-2018. Diabetes Technol Ther.

