# Supplementary material for: Psychoeducational Messaging to Reduce Alcohol Use for College Students With Type 1 Diabetes: Internet-Delivered Pilot Trial
Source: J Med Internet Res. 2021 Sep 30;23(9):e26418. doi: 10.2196/26418 (PMC8517820; doi:10.2196/26418)
Supplement: Multimedia Appendix 1 [file jmir_v23i9e26418_app1.docx]

**Multimedia Appendix 1.** Qualitative themes regarding intervention impressions and impact.

| **Theme and subtheme** | | **Illustrative quotes** |
| --- | --- | --- |
| **Information novelty and acquisition** | | |
|  | Verification of information gains after the intervention | - “As I filled out the section about how alcohol can effect certain aspects of diabetic care, I was upset to realize that I had never even learned about it. I figured it was bad for me, but I didn’t realize the extent.” - “I thought I knew pretty much everything there was to know about drinking with diabetes; however, I was wrong. I did not know the actual science behind it.” - “I knew that alcohol affected my blood sugar but I did not know exactly how it did. This video was very informative, and relayed in a very easy and simple way.” |
|  | Lack of information derived from medical providers | - “I do wish my doctors would have told me more to prepare me for turning 21 just so I’d have the information.” - “I had to ask, specifically, and multiple times for information on how to drink safely with diabetes. Even then I was not given all the information that I apparently needed...transition care was and is tough, and I am still very much trying to find my footing in the adult diabetes care world.” - “Honestly, the video provided me with more information than any of my endocrinologists have on the topic of drinking. I’ve been told that drinking is ‘just something I shouldn’t do’ with no further explanation...this went into greater depth.” |
|  | Sources of previously acquired information | - “I was reminded of why I do not drink mixers or wine anymore, due to the fact that the sugar in those specific drinks cause a spike in blood sugar, to which I correct, then my blood sugar drops fast. The end result is not worth the drink. I learned about this by looking at Google.” - “I went to a diabetes camp as a teenager and they had a panel of college kids come talk to us about drinking and then had an endo come talk about why your body reacted the way it does I found that very helpful and I feel like this video could provide that information for teens who didn’t get the education I did.” - “I was kind of disinterested at first, because this is a lot of information I have heard before. My health care team reiterates these guidelines every visit. However overall I think the video was well done!” |
| **Importance of personal safety and harm reduction** | | |
|  | Desire for harm reduction messages | - “I did not agree with the video’s message of just telling T1D’s to just not drink alcohol. Most college aged kids that I know would not [abstain] if told to, and straight up telling us not to drink is not going to stop us from drinking, it might even perpetuate us to drink more.” - “I don’t like though how the people at the end make it seem like you absolutely cannot drink. From talking to my doctor, if you want to drink, make sure to drink responsibly and proportion to your body.” - “I like how even though you are pushing for abstinence regarding alcohol, you provide facts and tips for those who choose to indulge regardless.” |
|  | Established safety practices | - “I am careful to only drink when my Dexcom is working, and always wear a medical ID bracelet (which I never do otherwise).” - “I have found that checking my blood sugar often, drinking light beers or using diet mixers, and only drinking with friends who are aware of my diabetes has allowed me to drink comfortably while in college.” - “Whenever I drink I carry my backpack with me and keep a glucagon in the same pocket always, and make sure that at least one of the people with me knows how to use a glucagon. I have people practice loading up a glucagon with old/expired glucagon so they actually know how to do it.” |
|  | Uptake of new safety practices after the intervention | - “The facts I learned from the video have definitely stuck with me and contribute to my decision to stop drinking alcohol, even just socially.” - “Last weekend before I went to bars, I thought about the video and though more diligently about my alcohol consumption and was more conscious about taking care of my diabetes.” - “I thought it was very educational and was glad I learned about the danger I’m putting myself in when I drink. I now know how to take care of myself and won’t ever push my limits on drinking.” |
| **Desire for affirming and relatable content** | | |
|  | Identification, or lack thereof, with patient experiences presented in the video | - “I can definitely relate to and have experienced many of the things said throughout the video. It was especially effective because they were coming from actual people with diabetes.” - “I think that this video gives a lot of great information, but is very bland and feels unrelatable...I have heard a lot of this information my whole life, it would resonate more with me if it weren’t so educational and more personal (stories, unscripted, mixture of info and personal experiences, actual faces instead of quotes and voices).” - “I agree with the statement ‘I feel as though diabetes has made me have to grow up at a young age...’ I have felt this way my whole life. When I have been ‘drunk’ and low, I did not experience the sweating and shaking low symptoms that I normally do, so I was able to relate my experience to this.” |
|  | Developmental appropriateness of video content | - “This video felt incredibly pertinent to me at the moment, as I am 22 and just starting to try to figure out how to drink with diabetes.” - “The video was effective in providing a lot of helpful information and making it relevant to the experiences of people with type one in college.” - “When students are in college, diabetes is the last thing we want to think about...I think if it was shorter and maybe texted or tweeted out to students in small portions, students would be more likely to watch.” |
|  | Provider gravitas | - “I appreciated that the Doctor was both pediatric and adult. It made me feel more comfortable that she knows a lot about the transition into adulthood.” - “I like this doctor, she seems trustworthy.” - “Like the fact that an endo comes straight out. Makes the video have authority.” |
|  | Video quality and professionalism | - “I really liked the information in the video and think it is something that is missing in diabetes education. However, I felt like the video could have been shorter and more professional.” - “This video seemed professional and seemed to provide me with accurate information.” - “I think the information was good but the acting was bad.” |
| **Stress and support in college** | | |
|  | Residual safety and self-management concerns | - “I haven’t drank in college at all, partly based on my lifestyle choices, but also because of the risks associated with T1D...I am now considering drinking, but concerns about the added risks from drinking with T1D still scare me.” - “It’s been hard to manage diabetes on top of everything else like school, classes, exams, clubs, activities, and more. I’ve felt diabetes burnout at college and lose the motivation to do simple things like check my blood sugar, which is something that has never happened to me before college.” |
|  | Importance of appropriate and supportive providers | - “It has been hard to find an endocrinologist near my school. Leading me to be without prescriptions for days at a time. Which has made my diabetes hard to manage.” - “Most of my information about diabetes care in general has been through education from...nurses/on site endocrinologists. It was an integral part of how I view my diabetes, my positive outlook on life, and my awareness of my disease.” - “I think there is definitely a lack of education in this area because doctors don’t want to talk to their patients about alcohol before going to college, because they are underage.” |
|  | Social connectedness and peer influence | - “Having a good group of friends has been the key to my success in being able to manage my diabetes while I am drinking.” - “I am one of only a few type one diabetics on my campus, and often times I do feel like I have to explain or even justify why I am carrying a juice at a party or why my insulin pump tubing is visible, which can be really frustrating.” - “I’ve always said no to drinking but recently have found myself in situations with new people where I want to feel included, so I’ve been trying to figure out the effects of alcohol on my blood sugars. I know that this isn’t always a good idea, but sometimes by desire to be ‘normal’ overpowers the part of me that knows I should just not drink.” |
