# Supplementary material for: Psychoeducational Messaging to Reduce Alcohol Use for College Students With Type 1 Diabetes: Internet-Delivered Pilot Trial
Source: J Med Internet Res. 2021 Sep 30;23(9):e26418. doi: 10.2196/26418 (PMC8517820; doi:10.2196/26418)
Supplement: Multimedia Appendix 2 [file jmir_v23i9e26418_app2.docx]

**Appendix 2 – Adjusted Results for Specific Items**

|  | **Main Effects Models** | | |  | **Interaction Models** | | |
| --- | --- | --- | --- | --- | --- | --- | --- |
|  | **Est.** | **95% CI** | **p-value** |  | **Est.** | **95% CI** | **p-value** |
| **Select Knowledge Items** ^a^ |  |  |  |  |  |  |  |
| ***Drinking alcohol causes the liver to release sugar into the bloodstream*** | | | | | | | |
| Peer vs Provider | 0.46 | (-0.22, 1.13) | 0.183 |  | 0.59 | (-0.27, 1.45) | 0.178 |
| Post-intervention vs Baseline | **0.81** | **(0.39, 1.22)** | **<.001** |  | **0.94** | **(0.31, 1.56)** | **0.003** |
| Follow-up vs Baseline | 0.12 | (-0.28, 0.51) | 0.563 |  | 0.17 | (-0.37, 0.71) | 0.531 |
| Peer * Post-intervention |  |  |  |  | -0.25 | (-1.07, 0.58) | 0.562 |
| Peer * Follow-up |  |  |  |  | -0.10 | (-0.88, 0.68) | 0.801 |
| ***It is easy to tell the difference between being drunk and having low blood sugar*** | | | | | | | |
| Peer vs Provider | -0.08 | (-1.01, 0.85) | 0.872 |  | 0.11 | (-0.88, 1.09) | 0.830 |
| Post-intervention vs Baseline | **0.59** | **(0.12, 1.06)** | **0.014** |  | **0.76** | **(0.16, 1.35)** | **0.012** |
| Follow-up vs Baseline | **1.10** | **(0.52, 1.68)** | **<.001** |  | **1.80** | **(0.81, 2.78)** | **<.001** |
| Peer * Post-intervention |  |  |  |  | -0.32 | (-1.28, 0.64) | 0.513 |
| Peer * Follow-up |  |  |  |  | **-1.23** | **(-2.46, 0.00)** | **0.049** |
| ***Drinking alcohol can prevent glucagon from doing its full job*** | | | | | | | |
| Peer vs Provider | -0.01 | (-1.57, 1.54) | 0.988 |  | -0.46 | (-1.75, 0.84) | 0.488 |
| Post-intervention vs Baseline | **2.73** | **(1.25, 4.20)** | **<.001** |  | **2.23** | **(0.39, 4.07)** | **0.017** |
| Follow-up vs Baseline | **1.34** | **(0.57, 2.10)** | **<.001** |  | **1.13** | **(0.03, 2.23)** | **0.044** |
| Peer * Post-intervention |  |  |  |  | 0.95 | (-2.02, 3.93) | 0.530 |
| Peer * Follow-up |  |  |  |  | 0.40 | (-1.12, 1.92) | 0.609 |
| ***Drinking alcohol can cause dehydration and make it harder to bring down my blood sugar*** | | | | | | | |
| Peer vs Provider | **1.51** | **(0.38, 2.65)** | **0.009** |  | **1.46** | **(0.15, 2.77)** | **0.029** |
| Post-intervention vs Baseline | **0.76** | **(0.13, 1.38)** | **0.017** |  | **0.91** | **(0.13, 1.70)** | **0.022** |
| Follow-up vs Baseline | **1.59** | **(0.73, 2.45)** | **<.001** |  | **1.51** | **(0.55, 2.46)** | **0.002** |
| Peer * Post-intervention |  |  |  |  | -0.46 | (-1.67, 0.75) | 0.454 |
| Peer * Follow-up |  |  |  |  | 0.42 | (-1.98, 2.83) | 0.729 |
| ***Drinking alcohol only has short-term effects (a few hours) on my blood sugar*** | | | | | | | |
| Peer vs Provider | 0.54 | (-0.26, 1.34) | 0.183 |  | 0.54 | (-0.40, 1.48) | 0.257 |
| Post-intervention vs Baseline | 0.51 | (-0.04, 1.05) | 0.067 |  | 0.57 | (-0.07, 1.20) | 0.079 |
| Follow-up vs Baseline | **0.76** | **(0.20, 1.32)** | **0.008** |  | **0.68** | **(0.00, 1.35)** | **0.049** |
| Peer * Post-intervention |  |  |  |  | -0.13 | (-1.25, 1.00) | 0.827 |
| Peer * Follow-up |  |  |  |  | 0.20 | (-0.98, 1.37) | 0.743 |
|  |  |  |  |  |  |  |  |
| **Select Attitude Items** ^b^ |  |  |  |  |  |  |  |
| ***College students with diabetes can drink if they are careful*** | | | | | | | |
| Peer vs Provider | 0.06 | (-0.60, 0.71) | 0.862 |  | 0.32 | (-0.47, 1.11) | 0.427 |
| Post-intervention vs Baseline | 0.11 | (-0.14, 0.37) | 0.376 |  | 0.31 | (-0.13, 0.74) | 0.173 |
| Follow-up vs Baseline | **0.41** | **(0.12, 0.70)** | **0.005** |  | **0.58** | **(0.08, 1.07)** | **0.023** |
| Peer * Post-intervention |  |  |  |  | -0.37 | (-0.88, 0.14) | 0.158 |
| Peer * Follow-up |  |  |  |  | -0.32 | (-0.91, 0.26) | 0.279 |
| ***It is easy to tell my friends I can't drink because of my diabetes*** | | | | | | | |
| Peer vs Provider | -0.04 | (-0.85, 0.76) | 0.915 |  | -0.07 | (-1.05, 0.90) | 0.883 |
| Post-intervention vs Baseline | **0.62** | **(0.19, 1.06)** | **0.005** |  | **0.64** | **(0.04, 1.24)** | **0.035** |
| Follow-up vs Baseline | 0.13 | (-0.29, 0.55) | 0.542 |  | 0.05 | (-0.52, 0.63) | 0.862 |
| Peer * Post-intervention |  |  |  |  | -0.04 | (-0.91, 0.83) | 0.926 |
| Peer * Follow-up |  |  |  |  | 0.16 | (-0.68, 1.00) | 0.717 |
|  |  |  |  |  |  |  |  |
| **Select Concern Items** ^c^ |  |  |  |  |  |  |  |
| ***The impact of alcohol on the accuracy of diabetes tests (for example, A1c blood tests)*** | | | | | | | |
| Peer vs Provider | -0.62 | (-1.36, 0.13) | 0.104 |  | -0.18 | (-0.97, 0.61) | 0.653 |
| Post-intervention vs Baseline | 0.25 | (-0.02, 0.51) | 0.065 |  | **0.56** | **(0.12, 1.00)** | **0.013** |
| Follow-up vs Baseline | 0.22 | (-0.07, 0.52) | 0.141 |  | **0.59** | **(0.17, 1.01)** | **0.006** |
| Peer * Post-intervention |  |  |  |  | **-0.61** | **(-1.13, -0.09)** | **0.022** |
| Peer * Follow-up |  |  |  |  | **-0.72** | **(-1.30, -0.14)** | **0.014** |
| ***The effectiveness of glucagon as a rescue medication, if I need it to treat a severe hypo*** | | | | | | | |
| Peer vs Provider | **-0.64** | **(-1.26, -0.02)** | **0.044** |  | -0.37 | (-1.12, 0.38) | 0.332 |
| Post-intervention vs Baseline | **0.46** | **(0.17, 0.75)** | **0.002** |  | **0.73** | **(0.30, 1.15)** | **<.001** |
| Follow-up vs Baseline | 0.24 | (-0.08, 0.56) | 0.147 |  | 0.36 | (-0.13, 0.84) | 0.149 |
| Peer * Post-intervention |  |  |  |  | -0.52 | (-1.10, 0.05) | 0.074 |
| Peer * Follow-up |  |  |  |  | -0.23 | (-0.87, 0.41) | 0.486 |
|  |  |  |  |  |  |  |  |
| **Select Intention Items** ^d^ |  |  |  |  |  |  |  |
| ***Tell my friends about the effect alcohol has on my diabetes*** | | | | | | | |
| Peer vs Provider | -0.01 | (-0.81, 0.79) | 0.983 |  | -0.45 | (-1.29, 0.38) | 0.289 |
| Follow-up vs Post-intervention | **-0.75** | **(-1.10, -0.40)** | **<.001** |  | **-1.20** | **(-1.78, -0.62)** | **<.001** |
| Peer * Follow-up |  |  |  |  | **0.89** | **(0.19, 1.58)** | **0.012** |
| ***Ask my doctor or health care provider about diabetes and alcohol use*** | | | | | | | |
| Peer vs Provider | 0.27 | (-0.46, 1.01) | 0.467 |  | -0.10 | (-0.93, 0.72) | 0.809 |
| Follow-up vs Post-intervention | -0.16 | (-0.52, 0.21) | 0.404 |  | -0.54 | (-1.09, 0.01) | 0.054 |
| Peer * Follow-up |  |  |  |  | **0.75** | **(0.03, 1.47)** | **0.042** |
|  |  |  |  |  |  |  |  |
| **Select Impression Items** ^e^ |  |  |  |  |  |  |  |
| ***The person in the video was knowledgeable about alcohol use*** | | | | | | | |
| Peer vs Provider | **-1.15** | **(-1.67, -0.63)** | **<.001** |  | **-1.22** | **(-1.75, -0.68)** | **<.001** |
| Follow-up vs Post-intervention | **-0.39** | **(-0.65, -0.12)** | **0.005** |  | **-0.46** | **(-0.83, -0.09)** | **0.015** |
| Peer * Follow-up |  |  |  |  | 0.14 | (-0.39, 0.67) | 0.608 |
| ***The person in the video was knowledgeable about diabetes*** | | | | | | | |
| Peer vs Provider | **-0.89** | **(-1.39, -0.39)** | **<.001** |  | **-1.00** | **(-1.50, -0.50)** | **<.001** |
| Follow-up vs Post-intervention | **-0.47** | **(-0.71, -0.22)** | **<.001** |  | **-0.58** | **(-0.91, -0.24)** | **<.001** |
| Peer * Follow-up |  |  |  |  | 0.21 | (-0.28, 0.70) | 0.400 |
| ***I could trust the person in the video*** | | | | | | | |
| Peer vs Provider | **-1.25** | **(-2.02, -0.48)** | **0.002** |  | **-1.25** | **(-2.10, -0.41)** | **0.004** |
| Follow-up vs Post-intervention | -0.15 | (-0.51, 0.22) | 0.426 |  | -0.15 | (-0.72, 0.42) | 0.599 |
| Peer * Follow-up |  |  |  |  | 0.01 | (-0.72, 0.74) | 0.979 |
| ***I could trust the information in the video*** | | | | | | | |
| Peer vs Provider | **-0.75** | **(-1.38, -0.11)** | **0.021** |  | **-0.81** | **(-1.48, -0.14)** | **0.017** |
| Follow-up vs Post-intervention | -0.30 | (-0.62, 0.02) | 0.064 |  | -0.37 | (-0.80, 0.05) | 0.088 |
| Peer * Follow-up |  |  |  |  | 0.13 | (-0.50, 0.77) | 0.679 |
| ***I would be willing to share this video with a friend/acquaintance who has T1D*** | | | | | | | |
| Peer vs Provider | **-0.93** | **(-1.83, -0.03)** | **0.042** |  | **-1.11** | **(-2.09, -0.13)** | **0.026** |
| Follow-up vs Post-intervention | -0.02 | (-0.42, 0.38) | 0.936 |  | -0.20 | (-0.78, 0.38) | 0.492 |
| Peer * Follow-up |  |  |  |  | 0.36 | (-0.44, 1.16) | 0.376 |
| ***I learned something I didn't already know*** | | | | | | | |
| Peer vs Provider | 0.41 | (-0.68, 1.50) | 0.462 |  | -0.17 | (-1.43, 1.08) | 0.785 |
| Follow-up vs Post-intervention | **-0.75** | **(-1.31, -0.20)** | **0.008** |  | **-1.36** | **(-2.25, -0.46)** | **0.003** |
| Peer * Follow-up |  |  |  |  | **1.17** | **(0.06, 2.27)** | **0.039** |

All models adjust for age at survey, age at diagnosis, sex, race/ethnicity, parent education, last hemoglobin A1c, and continuous glucose monitoring device use. Models for binge use additionally adjust for atypical events affecting the likelihood of drinking.

The ‘Peer*Time Interaction’ coefficient represents the difference in the change over time for the peer vs the provider arm; *this indicates if effect of the intervention differed by arm*. When not statistically significant at p<0.05, results from the ‘Main Effects’ column are preferred.

1. Knowledge items were modeled with a binomial distribution (correct vs not) though beta coefficients (not odds ratios) are shown.
2. Attitude items (scored from strongly disagree (1) to strongly agree (10)) were modeled with a normal distribution.
3. Concern items (scored from not concerned (0) to very concerned (6)) were modeled with a normal distribution.
4. Intention items (scored from definitely will not (1) to definitely will (8)) were modeled with a normal distribution.
5. Impression items (scored from strongly disagree (1) to strongly agree (10)) were modeled with a normal distribution.
